# Associations Among Sex, Cognitive Ability, and Autism Symptoms in Individuals with Down Syndrome

**DOI:** 10.1007/s10803-022-05779-6

**Published:** 2022-10-31

**Authors:** Laura del Hoyo Soriano, Audra Sterling, Jamie Edgin, Debra R. Hamilton, Elizabeth Berry-Kravis, Amanda Dimachkie Nunnally, Angela John Thurman, Leonard Abbeduto

**Affiliations:** 1 MIND Institute, University of California Davis, 2825 50th Street, Sacramento, CA USA; 2 Department of Psychiatry and Behavioral Sciences, University of California, Davis Health, Sacramento, CA USA; 3 Waisman Center, Department of Communication Sciences and Disorders, University of Wisconsin-Madison, Madison, WI USA; 4 Department of Psychology, University of Arizona, Tucson, AZ USA; 5 Department of Human Genetics, School of Medicine, Emory University, Atlanta, GA USA; 6 Departments of Pediatrics, Neurological Sciences and Biochemistry, Rush University Medical Center, Chicago, USA

**Keywords:** Autism, Down syndrome, Sex-differences, Females, Cognitive ability

## Abstract

This study explores sex-differences in (a) rates and profiles of autism symptoms as well as in (b) the contribution of intellectual quotient (IQ) to autism symptom presentation in Down syndrome (DS). Participants were 40 males and 38 females with DS, aged 6 to 23 years. Autism symptoms were rated through the Autism Diagnostic Observation Schedule-Second Edition (ADOS-2). Results show no sex differences in the ADOS-2 Calibrated Severity Scores (CSS). However, only females with DS who are classified as DS-Only have higher scores on verbal IQ than those classified as DS + autism. Furthermore, associations between IQ and all CSSs are found for females, but not for males. Findings suggest that verbal cognition may play differential roles for females and males with DS.

## Introduction

Down syndrome (DS) is the leading genetic cause of intellectual disability (ID), with an occurrence of 1 in 691 live births (Parker et al., 2010). DS is caused by the triplication of all (~ 95% of cases) or part of chromosome 21, resulting in a complex condition that affects physical, cognitive, and behavioral development (Sherman et al., 2007). A characteristic set of physical features (i.e., facial dysmorphology, a proportionally large tongue, low muscle tone, short stature) are associated with DS. In addition, individuals with DS are at elevated risk for several medical conditions (i.e., congenital heart defects, hearing and vision problems, sleep problems, low thyroid function, allergies, seizures; (Capone et al., 2006; del Hoyo Soriano, Rosser, et al., 2020; Esbensen et al., 2022; Rosser et al., 2018)). The cognitive phenotype associated with DS includes impairments beyond developmental-level expectations in language and communication, phonological processing, and verbal aspects of memory, as well as relative strengths in some nonverbal cognitive abilities (del Soriano et al., 2018; del Hoyo Soriano et al., 2020; Godfrey & Lee, 2018; Lanfranchi et al., 2010; Loveall et al., 2017; Pennington et al., 2003). Despite this characteristic phenotype, there is considerable variability in the presentation and severity of challenges observed among individuals with DS. This variability is associated with a constellation of environmental and biological factors (del Hoyo et al., 2016; del Soriano et al., 2021; Karmiloff-Smith et al., 2016; Thomas et al., 2020). In the study reported here, we examine the association of sex (as assigned at birth, hereafter simply “sex”) with variation in the behavioral phenotype of DS.

Sex differences have been reported across several domains for individuals with DS, including language, nonverbal cognition, and behavior. For example, scores on measures of expressive language (del Soriano et al., 2018), memory, executive functioning, and adaptive behavior (de Sola et al., 2015) are higher in females than in males with DS matched on mental age. Sex differences among individuals with DS have also been found in pragmatics, or the social uses of language (Lee et al., 2017), although not all such studies have reported sex differences (del Soriano et al., 2018; (Martin et al., 2017). Socioemotional difficulties have also been reported, with females with DS exhibiting higher levels of emotional challenges, such as worries and fears, unhappiness, emotional dependence and somatic symptoms, compared to males with DS (Achenbach et al., 2003; Jahromi et al., 2008; Nærland et al., 2017). In addition, some studies have suggested that males with DS exhibit more externalizing problems, such as aggressive behavior, compared to their female peers (van Gameren-Oosterom et al., 2013), although not all studies have found such differences (Dykens et al., 2002; Rice et al., 2015).

Beyond the challenges already noted, individuals with DS, on average, are also at greater risk than the general population for symptoms of autism (Channell et al., 2019; Nunnally et al., 2021; Reilly, 2009). (Note that throughout this paper” “autism” should be taken to refer to “autism spectrum disorder” in an attempt to avoid ablest language). In particular, the co-occurrence of autism among individuals with DS is currently estimated to be between 16% and 42% (DiGuiseppi et al., 2010; Nunnally et al., 2021; Oxelgren et al., 2017; Warner et al., 2017), which is much higher than the 2.3% observed in the general population (Maenner et al., 2021). However, it is important to recognize that these estimates are based primarily on symptoms observed on various autism screening tools. Moreover, even individuals with DS who do not have co-occurring autism display elevated rates of restrictive and repetitive behaviors and difficulties in social cognition, social communication, social awareness, and social motivation. Such behaviors are central to an autism diagnosis (Channell et al., 2015). Interestingly, individuals with DS with more limited cognitive or linguistic abilities appear to be especially likely to present with increased severity of autism symptoms (Channell et al., 2015; Reilly, 2009).

In the general population, sex is related to autism symptoms, with more males than females receiving an autism diagnosis (Halladay et al., 2015; Loomes et al., 2017; Maenner et al., 2020; Murphy et al., 2016). In the case of DS, however, the data regarding sex-related differences in autism symptoms or diagnosis have been inconsistent. Channell et al., (2019) examined the association between autism symptomatology, sex, and IQ in 203 individuals with DS between 6 and 25 years of age. This study found that autism symptoms, assessed via the Social Responsiveness Scale (SRS) (Constantino & Gruber, 2012), were negatively associated with IQ. Channell et al. also found no link between autism symptoms and sex in this sample. Similarly, Moss and colleagues (Moss et al., 2013) used the Social Communication Questionnaire (SCQ; (Rutter et al., 2003) in a sample of individuals with DS ages 4 to 39 years, also finding a lack of sex differences in autism symptoms. In contrast, Nærland et al., (2017) found that males with DS were more likely than females with DS to have scores in the clinical range on the SCQ. Nærland et al. also reported a correlation between severity of ID (determined by parent report on a formal cognitive assessment) and SCQ scores, such that more severe ID was associated with higher rates of autism symptoms.

In contrast to previous studies using informant-report screening measures to assess autism symptoms, we addressed sex differences in autism classifications and symptoms severity using a direct observational measure (i.e., Autism Diagnostic Observation Schedule, Second Edition (ADOS-2). We also focused on whether the relationship between verbal and nonverbal cognitive skills and autism symptomatology varies with sex among individuals with DS. This latter interest was motivated by several studies demonstrating an association between autism symptomatology and cognitive ability, both verbal and nonverbal, in individuals with DS (Channell et al., 2019; Nærland et al., 2017; Nunnally et al., 2021). However, whether these relationships differ for males and females with DS has previously been examined using only informant report measures of autism symptoms rather than direct observational measures as in the present study.

In the present study, we focused on a sample of verbal individuals with DS, ages 6 to 23 years. This sample overlaps considerably with that of Nunnally et al., (2021), who examined ADOS-2 classification rates, symptom profiles, and associated developmental domains. Nunnally et al. found that expressive language skills assessed through analysis of naturalistic language samples (Thurman et al., 2020) were related to ADOS-2 classification status. Group means in expressive language were lower for participants classified as co-occurring DS and autism (DS + AUT) than for participants classified as DS-only. However, nonverbal cognitive ability (assessed through growth scores on the Stanford-Binet Intelligence Scales, Fifth Edition; SB-5) was not related to ADOS-2 classification status. Nunnally et al. did not examine the role of sex on the occurrence or severity of autism symptoms classification rates or autism symptom severity. They also did not examine sex differences in the association between cognitive ability and autism symptoms. In the present study, we use SB-5 nonverbal and verbal IQ scores to measure the contributions of cognition to autism symptom presentation.

In summary, the aims of the present study were to examine sex-related differences in: (1) autism symptom presentation (i.e., symptom severity and diagnostic classification rates) and (2) the association between cognitive ability and autism symptom presentation in verbal individuals with DS ranging from 6 to 23 years of age. Investigations that consider the impact of sex on the presence of autism symptoms in individuals with DS can help provide additional insights into the mechanisms underlying such symptoms in individuals with DS. In addition, such research will ultimately improve interventions, which is of special interest given the high prevalence of autism symptoms among individuals with DS.

## Methods

### Data Source and Study Sample

A total of 78 participants (40 males, 38 females) with DS, with a mean age of 15.8 years (SD = 5.2), were included in the current study. Participants and measures reported here are a subset of a larger project evaluating the psychometric properties of various expressive language measures for individuals with ID (Abbeduto et al., 2020; Thurman et al., 2021). The age range of the larger project was designed to include individuals who would likely be able to meaningfully complete the language measures and exclude those who might display clinically significant signs of Alzheimer’s Disease. The focus of the larger project also necessitated recruiting only individuals capable of producing multiword utterances at least occasionally (according to caregiver report). The wide age and ability level range are well suited to the aims of the larger study, however, the generalizability of our findings is limited to verbal individuals with DS in the present study.

The following additional inclusion criteria were followed in the larger project as well as in the current study: First, all participants provided medical documentation of DS (i.e., trisomy 21 or translocation) without mosaicism, and all met criteria for intellectual disability ID (i.e., IQ < = 70). In addition, based on caregiver report: Participants with DS (1) used speech as their primary mode of communication; (2) spoke English as their primary language; (3) did not have serious or uncorrected hearing or visual impairment; (4) were not enrolled in a clinical trial or experienced significant medication, treatment, or educational changes during the 8 weeks prior to the initial testing visit. Furthermore, for the current study, participants who had missing or incomplete data on the ADOS-2 or the SB-5 were excluded as well. Note that the sample for the present study was the same as that of Nunnally et al., (2021) except for the exclusion from the present study of five participants who did not have complete data available on the SB-5.

The study was conducted in accordance with the ethical standards of the relevant national and institutional committees on human experimentation and with the Helsinki Declaration of 1975, as revised in 2008. Each site obtained their Institutional Review Board’s (IRB) approval to conduct the project. Participants were recruited through clinics, community events/referral, conferences, advertisements, internet postings, participation in past research projects, and from university research registries. The participating caregiver/legal guardian provided written consent and participants with DS provided verbal assent before data collection. All data for the present study were collected at the initial visit of the Expressive Language Sampling (ELS) project.

## Measures

### The Autism Diagnostic Observation Schedule, Second Edition (ADOS-2):

The ADOS-2, a semi-structured standardized play-based assessment that measures autism symptoms, was administered by research-reliable examiners. Modules 1, 2 or 3 of the ADOS-2 were administered to participants as a function of their verbal skills following standard ADOS-2 guidelines: participants with limited speech received Module 1 (n = 6), those with phrase speech up to fluent speech received Module 2 (n = 38), and those who produced a range of flexible sentence types, providing language beyond the immediate context, and describing logical connections within a sentence, received Module 3 (n = 34). Of the 38 females in the sample, 3 received Module 1, 15 received Module 2 and 20 received Module 3. In comparison, out of the 40 males in the sample, 3 received Module 1, 23 received Module 2 and 14 received Module 3. Module 4 was not administered to any participants given the level of functional skills required for that module. Because calibrated severity scores (CSS) have a more uniform distribution across developmental groups and are less influenced by participant demographics than raw scores (Gotham et al., 2009), the following scores were calculated for the current study: ADOS-2 diagnostic classification rates, Overall-(CSS), Social Affect (SA)-CSS, and Restricted and Repetitive Behavior (RRB)-CSS (Gotham et al., 2009; Lord et al., 2012). Both the Overall-CSS and SA-CSS are assessed using a 10-point scale, whereas the RRB-CSS entails using a 7-point scale across a 10-point scale range in which 2, 3, and 4 point scores are not possible to obtain (Hus & Lord, 2014). In accordance with standardized ADOS-2 procedures, participants who earn an Overall-CSS score of 4 or higher meet criterion for an autism classification (Lord et al., 2012). Note that for those participants older than the norming sample of the ADOS-2, the upper age limit of the CCS norming tables was used to compute CCSs. Inter-examiner reliability was assessed for 13 administrations, using videotape review. Mean percent agreement, relative to consensus scores achieved through group discussion, was 86% when all items were considered and 87% when only algorithm items were considered.

### Stanford-Binet Intelligence Scales, Fifth Edition (SB-5):

Cognitive functioning was assessed using the Stanford-Binet Intelligence Scales, Fifth Edition (SB-5; (Roid, 2003). Because previous research in ID has found that meaningful variation in cognitive ability on standardized IQ tests is lost when converting raw scores to IQ scores (i.e., substantial floor effects), we computed deviation scores for Non-Verbal IQ (NVIQ), and Verbal IQ (VIQ) following procedures outlined by Sansone et al. (Sansone et al., 2014). The use of the deviation z-score method rectifies this problem, and accounts for significant variance in criterion validation measures, above and beyond the usual IQ scores.

### Statistical Analyses

We first present descriptive statistics regarding age, NVIQ and VIQ for males and females separately. Next, we address whether there were sex-related differences in these variables through one-way ANOVAs, along with Eta-squared effect sizes (point estimates), with the 95% confidence interval, to decide whether we needed to control for any of the variables in the primary analyses. We also addressed whether there were age-related differences between participants who met/did not meet autism classification standards on the ADOS-2 to decide whether age needed to be included in our models.

To address our first aim, the male:female ratio for autism classifications was determined (i.e., the number of males and females who earned an autism classification on the ADOS-2). For this purpose, crosstabulations were performed, including sex (male/female) and autism classification (present/absent). We also computed a nominal by interval “risk” statistic to address the odds ratio male:female. In addition, crosstabulations were conducted to compare the frequency of classification in Module 1, Module 2 and Module 3 (columns) in males vs. females (row). Additionally, stratified distributions for CSS on the SA and RRB subdomains were computed for males and females of the DS + AUT group as well as for males and females for the entire sample (DS + AUT and DS-only). One-way ANOVAs were conducted, and effect sizes estimated to determine whether sex-related differences were present in SA-CSS and RRB-CSS in the DS + AUT group as well as in the entire sample (DS + AUT and DS-only).

To address our second aim, first, one-way ANOVAs were performed separately for NVIQ, and VIQ as dependent variables and autism classifications (i.e., was/was not classified as autism) for the males and for the females. Eta-squared effect sizes were also calculated (point estimates), along with the 95% confidence interval.

Second, linear regression models were performed to assess the association between cognitive ability (NVIQ, and VIQ) and autism severity (Overall-CSS, SA-CSS and RRB-CSS) in males and females separately. This approach allowed us to analyze the impact that cognitive ability had on autism severity for each sex. NVIQ, and VIQ were included as independent variables and Overall-CSS, SA-CSS and RRB-CSS scores were used as dependent variables, each in its separate model. Therefore, we conducted a total of 6 regression models divided in two sets of three analyses. In the first model of the first set of analyses, we introduced the NVIQ as independent variable and the Overall-CSS as the dependent variable. In the second model of the first set, the same independent variable was used but the SA-CSS was the dependent variable. In the third model of the first set, the same independent variable was used, with the RRB-CSS as the dependent variable. The second set of regression models followed the same procedures but instead of NVIQ, VIQ was the independent variable.

Third, we determined whether the association between cognitive ability and autism symptom severity differed between males and females, the interaction between sex and cognitive ability was analyzed. For this purpose, we computed a centered IQ for each scale (e.g., NVIQ - mean NVIQ, VIQ - mean VIQ). We then multiplied each value by the sex indicator to create an interaction variable (i.e., the “Interaction NVIQ” and the “Interaction VIQ”) to include in the models as a predictor. Overall-CSS, SA-CSS, and RRB-CSS were used as dependent variables. Again, we conducted a total of six regression models divided in two sets of analyses in the entire sample (i.e., males + females). In the first set of regression models, we introduced NVIQ, sex and the Interaction NVIQ as independent variables and the Overall-CSS as the dependent variable. In the second model, the same independent variables were used but the SA-CSS was the dependent variable. Finally, in the third model, the same independent variables were used, with the RRB-CSS as the dependent variable. The second set of regression models followed the same procedures but instead of NVIQ, VIQ was the independent variable along with sex and the Interaction VIQ.

Because multiple variables were included in each model, False Discovery Rate (FDR) corrections were applied to each of set of analyses in accordance with procedures outlined by Benjamini (Benjamini & Yekutieli, 2001).

All analyses were performed first in the entire sample (DS + AUT and DS-only), then only in participants who earned an autism classification (i.e., the DS + AUT subsample).

## Results

### General Characteristics of Participants and Potential Confounders

Table 1 presents descriptive statistics for the variables of interest, as well as participant demographics, stratified by sex. [Insert Table 1 here] Regarding potential confounders, one-way ANOVAs revealed no sex differences in NVIQ (F = 1.27, p = 0.26, PE = 0.02, CI = 0.00,0.11), VIQ (F = 0.24, p = 0.62, PE = 0.00, CI = 0.00,0.07), or age (F = 2.06, p = 0.16, PE = 0.03, CI = 0.00,0.13). Age was also not different between those who were/were not classified as autism on the ADOS-2 (F = 1.05, p = 0.31, PE = 0.01, CI = 0.00,0.10).

**Table 1:** Participant demographics and descriptive statistics for the variables of interest

	Overall Sample			Females			Males		
	**N**	**Frequency**	**%**	**N**	**Frequency**	**%**	**N**	**Frequency**	**%**
**Race**	69			34			35		
*African American/Black*		2	2.6		1	2.6		1	2.5
*Asian/Pacific* *Islander*		2	2.6		1	2.6		1	2.5
*White*		51	65.4		25	65.8		26	65.0
*Other*		1	1.3		1	2.6		0	0.0
**Ethnicity**									
*Hispanic/Latino*		13	16.7		6	15.8		7	17.5
**Yearly Income** *Less than 25,000* *25,000–50,000* *50,000–75,000* *75,000–100,000* *100,000–150,000* *150,000–250,000* *Over 250,000*	75	6 16 14 9 14 12 4	7.7 20.5 17.9 11.5 17.9 15.4 5.1	37	4 5 8 6 7 5 2	10.5 13.2 21.1 15.8 18.4 13.2 5.3	38	2 11 6 3 7 7 2	5.0 27.5 15.0 7.5 17.5 17.5 5.0
	**N**	**Mean**	**SD**	**N**	**Mean**	**SD**	**N**	**Mean**	**SD**
**Age (years)**	78	15.84	5.21	38	16.70	4.91	40	15.02	5.41
**Cognitive ability**	78			38			40		
Nonverbal VIQ		49.81	12.15		51.39	10.98		48.40	13.13
Verbal IQ		40.68	13.10		41.44	13.63		39.96	12.72
**ADOS-2** ^**1**^	78			38			40		
Overall- CSS^2^		3.40	2.13		3.24	2.09		3.55	2.18
SA- CSS^3^		3.92	2.12		3.68	1.95		4.15	2.27
RRB- CSS^4^		4.37	2.47		4.37	2.36		4.38	2.60
Meet autism criteria		**Frequency** 31	**%** 39.74%		**Frequency** 14	**%** 36.84%		**Frequency** 17	**%** 42.5%

Footnotes: ^1^Autism Diagnostic Observation Schedule, Second Edition, ^2^ Overall-Calibrated Severity Score, ^3^ Social Affective-Calibrated Severity Score, ^4^ Restricted and Repetitive Behavior-Calibrated Severity Score. Non-significant differences were found between males and female in terms of age, cognitive ability, or ADOS-2 scores

### Autism Classification and Symptom Severity as Function of Sex

We found that 14 of 38 (36.8%) females were classified as DS + AUT, whereas 17 of 40 (42.5%) males were classified as DS + AUT. The risk estimate in terms of odds ratios analyses indicated a 1.3 times greater likelihood of being classified as having autism for males than for females (OR = 1.3; 95% CI = 0.5, 3.1). However, a one-way ANOVA showed no male-female difference in the Overall-CSS (F = 0.42, p = 0.52, PE = 0.01, CI = 0.00,0.08), SA-CCS (F = 0.94, p = 0.34, PE = 0.01, CI = 0.00,0.09) or RRB-CSS (F = 0.00, p = 0.99, PE = 0.00, CI = 0.00,0.00) for the sample as a whole (DS + AUT and DS-only). The ANOVA results were similar when the analyses were repeated on only the DS + AUT participants (in this case, all p-values were > 0.6).

### Relationship Between Cognitive Ability and Autism Classification and Symptom Severity as a Function of Sex

One-way ANOVAs were performed separately on the data for males and females. For the female participants, there was a significant difference in the VIQs (F = 11.29, p = 0.002) but not in the NVIQs (F = 2.78, p = 0.1) between those classified as DS-Only and those classified as DS + AUT. In contrast, for the male participants, there were no significant differences on the IQ measures between the participants classified as DS-Only and those classified as DS + AUT (VIQ: F = 0.8, p = 0.38; NVIQ: F = 0.17, p = 0.68).

As seen in Table 2, for each one-point increase in NVIQ, there was a decrease in the ADOS-2 Overall-CSS of 0.08 for females (p = 0.02) and a decrease 0.04 for males (p = 0.14), with the difference in the strength of the association for males and females failing to reach statistical significance (p = 0.36). For each one-point increase in NVIQ, there was a decrease in the SA-CSS of 0.06 for females (p = 0.04) and a decrease of 0.04 for males (p = 0.11), with the difference between males and females in the strength of this association not significant (p = 0.64). For each one-point increase in NVIQ, there was a decrease in the RRB-CSS of 0.06 for females (p = 0.10), but an increase of 0.01 for males (p = 0.76); again, however, the strength of this association was not significantly different for males and females (p = 0.13). [Insert Table 2 here]

**Table 2:** Relationship Between the SB-5 Intelligence Scale Nonverbal IQ Scale and the ADOS-2 as a function of sex

Predictor:	Dependent variable:	N	Unstandardized Coefficients βStd. Error		Standardized Coefficients Beta	t	p value
Nonverbal IQ (males)	ADOS-2^1^ Overall- CSS^2^	40	− 0.04	0.03	− 0.24	-1.51	0.14
Nonverbal IQ (females)	ADOS-2^1^ Overall- CSS^2^	38	− 0.08	0.03	− 0.43	-2.47	0.01*
Nonverbal IQ -Sex interaction	ADOS-2^1^ Overall- CSS^2^	78	0.04	0.04	0.16	0.92	0.36
Nonverbal IQ (males)	ADOS-2^1^ Social Affect -CSS^2^	40	− 0.05	0.03	− 0.26	-1.65	0.11
Nonverbal IQ (females)	ADOS-2^1^ Social Affect -CSS^2^	38	− 0.06	0.03	− 0.36	-2.07	0.04*
Nonverbal IQ -Sex interaction	ADOS-2^1^ Social Affect -CSS^2^	78	0.02	0.04	0.08	0.47	0.64
Nonverbal IQ (males)	ADOS-2^1^ Restricted and Repetitive Behavior-CSS^2^	40	0.01	0.03	0.05	0.31	0.76
Nonverbal IQ (females)	ADOS-2^1^ Restricted and Repetitive Behavior-CSS^2^	38	− 0.06	0.04	− 0.31	-1.69	0.09
Nonverbal IQ -Sex interaction	ADOS-2^1^ Restricted and Repetitive Behavior-CSS^2^	78	0.07	0.05	0.28	1.52	0.13

Footnotes: ^1^Autism Diagnostic Observation Schedule Second Edition, ^2^Calibrated Severity Score, IQ deviation z-score for Non-Verbal IQ were used following procedures outlined by Sansone et al., 2014. * p ≤ 0.050, ** p ≤ 0.010, *** p ≤ 0.005

Regarding the role of VIQ (Table 3), for each one-point increase in VIQ, there was a decrease in the ADOS-2 Overall-CSS of 0.09 for females (p < 0.001) and 0.05 for males (p = 0.06), with difference in strength of association between males and females not reaching statistical significance (p = 0.28). For each one-point increase in VIQ, there was a decrease in the SA-CSS of 0.08 for females (p = 0.001) and 0.05 for males (p = 0.06), but the difference between males and females in the strength of this association was not significant (p = 0.45). For each one-point increase in VIQ, there was a decrease in the RRB-CSS of 0.09 for females (p = 0.003) and an increase of 0.01 for males (p = 0.74), with a significant difference between males and females in the strength of this association (p = 0.02). [Insert Table 3 here]

**Table 3:** Relationship Between the SB-5 Intelligence Scale Verbal IQ Scale and the ADOS-2 as a function of sex

Predictor:	Dependent variable:	N	Unstandardized Coefficients βStd. Error		Standardized Coefficients Beta	t	p value
Verbal IQ (males)	ADOS-2^1^ Overall- CSS^2^	40	− 0.05	0.03	− 0.30	-1.90	0.06
Verbal IQ (females)	ADOS-2^1^ Overall- CSS^2^	38	− 0.09	0.02	− 0.54	-3.76	< 0.001***
Verbal IQ-Sex interaction	ADOS-2^1^ Overall- CSS^2^	78	0.04	0.03	0.16	1.09	0.28
Verbal IQ (males)	ADOS-2^1^ Social Affect -CSS^2^	40	− 0.05	0.03	− 0.30	-1.92	0.06
Verbal IQ (females)	ADOS-2^1^ Social Affect -CSS^2^	38	− 0.08	0.02	− 0.49	-3.37	0.001**
Verbal IQ-Sex interaction	ADOS-2^1^ Social Affect -CSS^2^	78	0.03	0.03	0.11	0.77	0.45
Verbal IQ (males)	ADOS-2^1^ Restricted and Repetitive Behavior-CSS^2^	40	0.01	0.03	0.05	0.33	0.74
Verbal IQ (females)	ADOS-2^1^ Restricted and Repetitive Behavior-CSS^2^	38	− 0.09	0.03	− 0.46	-3.02	0.003**
Verbal IQ-Sex interaction	ADOS-2^1^ Restricted and Repetitive Behavior-CSS^2^	78	0.10	0.04	0.36	2.35	0.021*

Footnotes: ^1^Autism Diagnostic Observation Schedule Second Edition, ^2^Calibrated Severity Score, IQ deviation z-score for Verbal IQ were used following procedures outlined by Sansone et al., 2014. * p ≤ 0.050, ** p ≤ 0.010, *** p ≤ 0.005

In the secondary analyses, in which we repeated all the previous analyses regression but only for participants who met criteria for autism (i.e., the DS + AUT subsample), none of the relationships between the IQ variables and the ADOS-2 variables were significant.

## Discussion

The current study was designed to examine sex-related differences in autism symptom presentation (i.e., classification status and symptom severity) and in the association between cognitive ability and autism symptom presentation in children, adolescents, and young adults with DS. First, no significant sex-related differences were observed in ADOS-2 classifications or severity scores. There was a significant difference, however, in VIQ between females with DS who were classified as DS-Only and those classified as DS + AUT on the ADOS-2, with the former having lower scores. No such difference was observed for the males. In addition, several significant associations between VIQ and NVIQ and ADOS-2 symptom severity were observed for the females, such that lower IQs were related to higher autism symptom severity. None of these associations between IQ and autism symptoms were found for the males. Nonetheless, the difference between males and females in the strength of these associations was significant only for the association between VIQ and RBB-CSS.

Our finding of no sex difference in the ADOS-2 diagnostic classification rates or symptom severity scores are largely consistent with previous research on individuals with ID. Although there is a 4:1 ratio of males to females in terms of nonsyndromic cases of autism, that ratio is reduced dramatically (i.e., 1.7:1) when one considers only those individuals who also have co-occurring ID (Baird et al., 2006; Fombonne, 2005; Kreiser & White, 2014; Rea et al., 2022). In addition, our results are in line with two previous studies conducted in DS that failed to find sex differences in scores on informant report screening tools, such as the SRS and SCQ (Channell et al., 2019; Moss et al., 2013), but not with results from Nærland et al., (2017), who reported sex-related differences on the SCQ. The present results thus provide additional support to previous research showing no sex differences in autism symptom co-occurrence among individuals with DS, but for the first time using a gold standard direct observation measure (i.e., ADOS-2).

Although there were no sex differences in autism classification rates or symptom severity scores, there were sex differences in the associations between cognitive ability and autism symptom presentation (significant only for females). Note, however, that only the association between VIQ and the RBB-CSS was significantly greater in magnitude for females than for males. This last result suggests that, in our sample of participants, the role that verbal cognitive ability plays in restrictive and repetitive behaviors is greater for females than for males with DS. This finding is of special interest as it may suggest sex differences in the types of RRBs observed and/or in the factors influencing the occurrence of these behaviors. For example, other studies have posited that RRBs may decline as gains are made in functional skills, such as language (Larkin et al., 2017; Ray-Subramanian & Ellis Weismer, 2012; Thurman et al., 2015). At the same time, other factors are also known to contribute to the presence of RRBs, such as anxiety or executive functioning skills (Leekam et al., 2011; Oakes et al., 2016). In addition, the correlates of RRBs are known to vary as a function of RRB type (Bishop et al., 2007; Gabriels et al., 2005; Miguel et al., 1997; Oakes et al., 2016). Elucidating sex differences in the nature of RRBs in DS will be an important next step in understanding autism symptomatology in individuals with DS. In addition, it is important to note that the SB-5 may not reflect the verbal and communication abilities that are displayed by individuals with DS in naturalistic contexts. Moreover, the range of skills aggregated in the SB-5 verbal IQ is quite broad and includes the verbal components of fluid reasoning, knowledge, quantitative reasoning, visual-spatial processing and working memory. It is possible that different verbal components are differentially related to autism symptoms. In our previous study (Nunnally et al., 2021), we saw that structural language skills (i.e., lexical diversity and syntactic complexity), measured using expressive language sampling procedures, differentiated participants who were classified as DS + AUT from those classified as DS-only. In the present study, we did not examine associations involving the separate components of verbal or nonverbal IQ. Future studies are planned to explore sex differences in the association between verbal skills and autism symptoms as a function of measurement approach and domain of skill assessed.

In females, we also observed stronger associations between verbal cognition and autism symptom domains (social affective and restrictive and repetitive behaviors) compared to those observed between nonverbal cognitive ability and those same domains. Indeed, when comparing females with DS who were classified as DS-Only and those classified as DS + AUT on the ADOS-2, there were significant differences in verbal cognitive ability but not in nonverbal cognition. This finding is consistent with the hypothesis that the role that ID plays in autism symptoms for females with DS might be especially relevant when verbal components of cognitive function are considered. In contrast, autism symptoms in males with DS may not be as closely tied to cognitive ability (whether verbal and nonverbal) as often assumed. These findings could reflect biases in measurement tools designed to assess autism characteristics or in the ability of DS females with higher IQs to compensate for their autism symptoms, similar to findings for autism in individuals without ID (Rea et al., 2022; Robinson et al., 2013). It could also be that cognitive ability is more protective against autism symptoms in females than in males with DS. Additional research, however, is needed to examine these and other possible explanations using larger samples and multiple measures of the constructs of interest.

## Limitations

There are several limitations of the current study. First, we only used the ADOS-2 to consider autism symptom presentation; therefore, it is unknown whether the findings reported here are replicable when using other diagnostic measures. A comprehensive autism diagnosis requires a detailed developmental history and more information than is provided by the ADOS-2 alone to determine the extent to which individuals meet DSM-5 diagnostic criteria and to consider whether symptoms are better explained by other factors (e.g., presence of ID or other co-occurring conditions such as ADHD or anxiety). Second, we included only participants who produced at least some multi-word utterances because this was a criterion for inclusion in the larger project from which the current data were extracted. As a result, more severely impaired individuals, particularly those who would be considered minimally verbal, were not represented in the current study. Consequently, the generalizability of the present results is limited accordingly. Relatedly, those few participants who were administered Module 1 are youth whose parents reported them to produce at least some multi-word phrases, which is not the Module 1 criterion (i.e., pre-verbal/single words); however, these same participants only produced single words during the ADOS-2 assessment and thus, our screening on exclusion criteria was imperfect. Third, the norming age of the ADOS-2 only goes up to 14 years of age for Module 1 and up to 16 years of age for Modules 2 and 3 for severity scores. Therefore, a very large part of our sample was not in the norming range, which limits the interpretation of our data. As stated in the methods, for those participants older than the norming sample, the upper age limit of the CCS norming tables was used to compute CCSs. Finally, although chronological age was not related to autism symptom presentation nor to IQ scores or sex, we must acknowledge the wide age range of the current study. For this reason, future studies should examine sex-related differences in autism symptoms in DS with a larger sample and through stratified analyses by age groups to further understand the contribution of age.

## Conclusion

Findings from the current study support previous research conducted in DS (Channell et al., 2019; Moss et al., 2013) and ID in general (Rea et al., 2022), showing no sex differences in the prevalence of an autism classification and autism symptom severity, as measured by the ADOS-2, a gold standard diagnostic instrument. We emphasize, however, the contribution of verbal cognitive ability to the classification of autism as well as the contribution of both verbal and nonverbal cognitive ability to autism symptom severity in our female subsample of participants with DS but not in the male group, suggesting a sex-specific role that cognitive ability plays in the observation of autism symptoms for individuals with DS. Such results are of special interest, as this could impact interventions in DS in terms of adopting a sex-based approach. To conclude, this study contributes to the field by showing no sex-related differences in the prevalence and presentation of autism in individuals with DS. Importantly, it is the first study to analyze the link between cognitive ability and autism in DS. Additionally, it also demonstrates the need for more research exploring the sex-specific role that IQ plays in autism classification and symptom severity among individuals with DS.

## Data Availability

The datasets used for the present paper can be made available upon a reasonable request to the corresponding author.
